# P-1584. Impact of SARS-CoV-2 Infection (COVID-19) by Trimester on Severity of Illness and Pregnancy Outcomes: A Retrospective Cohort Study

**DOI:** 10.1093/ofid/ofaf695.1763

**Published:** 2026-01-11

**Authors:** Muzna Lone, Syeda Fatima Shariq, Muhammad Shuaib Afzal, Aimon Malik, Jameela Ali Al Ajmi, Adeel A Butt

**Affiliations:** Hamad Medical Corporation, Doha, Ar Rayyan, Qatar; Hamad Medical Corporation, Doha, Ar Rayyan, Qatar; Hamad Medical Corporation, Doha, Ar Rayyan, Qatar; Hamad Medical corporation, Doha, Ad Dawhah, Qatar; Hamad Medical Corporation, Doha, Ar Rayyan, Qatar; Hackensack Meridian JFK University Medical Center , Edison, NJ

## Abstract

**Background:**

The impact of COVID-19 on pregnancy outcomes remains controversial, with studies reporting conflicting results. Limited data exist on whether the timing of infection explains these discrepancies. The aim of this study is to determine whether COVID-19 infection acquired during different trimesters in pregnancy was associated with varying severity of illness and pregnancy outcomes.

**Figure:**
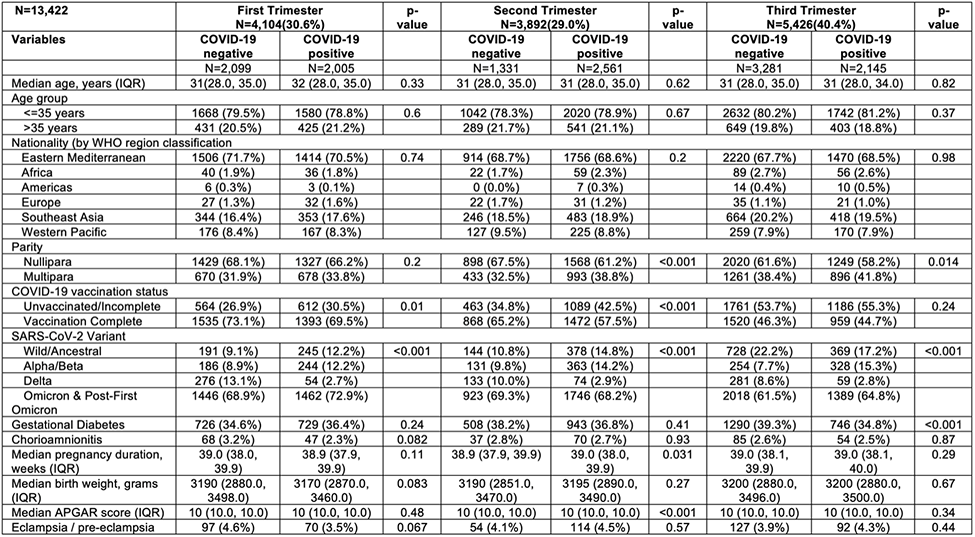
Characteristics of Pregnant Women by Trimesters of Pregnancy N, Number; IQR, Inter Quartile Range; WHO, World Health Organization, APGAR score: Appearance, Pulse, Grimace, Activity and Respiration

**Figure:**
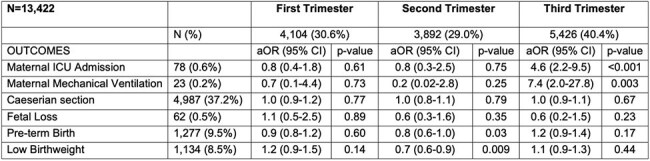
The association of COVID-19 infection during pregnancy and the risk of severity of illness and pregnancy outcomes, by trimester of COVID-19 infection. (Multivariable logistic regression model) N, Number; ICU, Intensive Care Unit; aOR, adjusted odds ratios The models have been adjusted for age group, parity status, vaccination status, SARS-CoV-2 variants, eclampsia/pre-eclampsia, gestational diabetes mellitus and chorioamnionitis.

**Methods:**

The study was conducted in Hamad Medical Corporation (HMC), Qatar. HMC handled about 84% obstetric cases in the country from 2019-2022. Retrospective data of pregnant women between January 1, 2020 and May 31, 2024 were retrieved from electronic medical records. Singleton pregnancies with a first RT-PCR confirmed infection were 1:1 exactly matched on 5-year age groups, WHO nationalities, gravida, COVID-19 test timing and vaccination status with uninfected women with at least 1 negative and no positive test during pregnancy. Final dataset comprised 6,711 COVID-19 positive and 6,711 matched COVID-19 negative pregnancies. Outcomes included maternal intensive care unit (ICU) admission, maternal mechanical ventilation, birth by cesarean section,fetal loss, preterm birth (birth before 37 weeks) and low birthweight (birth weight less than 2500 grams). Adjusted odds ratios were calculated stratified by infection trimester.

**Results:**

Baseline characteristics were similar across groups. Vaccination rates were similar in the third trimester but lower in the COVID-19 positive group during the first (69.5% vs.73.1%) and second trimesters (57.5% vs. 65.2%) compared to uninfected group. COVID-19 infection in the third trimester was associated with higher odds of ICU admission (aOR 4.6, 95% CI 2.2-9.5, p< 0.001), and mechanical ventilation (aOR 7.4, 95% CI 2.0-27.8, p=0.003), but not in the first or the second trimesters. COVID-19 infection in any trimester was not associated with higher odds of cesarean birth, fetal loss, preterm birth or low birth weight.

**Conclusion:**

COVID-19 infection acquired during the third trimester of pregnancy was associated with higher rates of maternal ICU admission and mechanical ventilation. However, infection during any trimester was not consistently associated with fetal loss. Second-trimester infection was linked to lower odds of preterm birth and low birthweight.

**Disclosures:**

All Authors: No reported disclosures

